# Validity of Simplified 3′-Deoxy-3′-[^18^F]Fluorothymidine Uptake Measures for Monitoring Response to Chemotherapy in Locally Advanced Breast Cancer

**DOI:** 10.1007/s11307-012-0547-1

**Published:** 2012-03-06

**Authors:** Mark Lubberink, Wieteke Direcks, Jasper Emmering, Harm van Tinteren, Otto S. Hoekstra, Jacobus J. van der Hoeven, Carla F. M. Molthoff, Adriaan A. Lammertsma

**Affiliations:** 1 Department of Nuclear Medicine & PET Research, VU University Medical Centre, Amsterdam, The Netherlands; 2 Nuclear Medicine & PET, Uppsala University, Uppsala, Sweden; 3 Department of Medical Oncology, Medical Centre Alkmaar, Alkmaar, The Netherlands; 4 Netherlands Cancer Institute, Amsterdam, The Netherlands; 5 PET Centre, Uppsala University Hospital, 751 85 Uppsala, Sweden

**Keywords:** PET, FLT, SUV, Modelling, Response monitoring

## Abstract

**Purpose:**

Positron emission tomography using 3′-deoxy-3′-[^18^F]fluorothymidine ([^18^F]FLT) has been suggested as a means for monitoring response to chemotherapy. The aim of this study was to evaluate the validity of simplified uptake measures for assessing response to chemotherapy using [^18^F]FLT in locally advanced breast cancer (LABC).

**Procedures:**

Fifteen LABC patients underwent dynamic [^18^F]FLT scans both prior to and after the first cycle of chemotherapy with fluorouracil, epirubicin or doxorubicin, and cyclophosphamide. The net uptake rate constant of [^18^F]FLT, *K*_*i*_, determined by non-linear regression (NLR) of an irreversible two-tissue compartment model was used as the gold standard. In addition to Patlak graphical analysis, standardised uptake values (SUV) and tumour-to-whole blood ratio (TBR) were used for analysing [^18^F]FLT data. Correlations and relationships between simplified uptake measures and NLR before and after chemotherapy were assessed using regression analysis.

**Results:**

No significant differences in both pre- and post-chemotherapy relationships between any of the simplified uptake measures and NLR were found. However, changes in SUV between baseline and post-therapy scans showed a significant negative bias and slope less than one, while TBR did not.

**Conclusions:**

In LABC, TBR instead of SUV may be preferred for monitoring response to chemotherapy with [^18^F]FLT.

## Introduction

The positron emission tomography (PET) tracer 3′-deoxy-3′-[^18^F]fluorothymidine ([^18^F]FLT) [1], a thymidine analogue that is a marker of tumour proliferation, is a promising tracer for monitoring treatment response and predicting outcome. It has been shown that [^18^F]FLT uptake strongly correlates with the proliferation index as measured by Ki-67 immunohistochemistry in lung and breast tumours [2], as well as with thymidine kinase-1 expression in lung tumours [3].

The gold standard for analysing tracer uptake in tissue is by non-linear regression (NLR) of operational equations based on compartmental models. In case of [^18^F]FLT, a two-tissue compartment model is used and, in general, it is assumed that phosphorylated tracer is irreversibly trapped [4–10]. When scan durations longer than 60 min are used, dephosphorylation can no longer be neglected and a two-tissue reversible compartment model may be preferred [8]. Alternatively, a basis function approach, not requiring prior assumptions about the exact underlying compartment model, can be used [11]. A good correlation between net uptake rate *K*_*i*_ of [^18^F]FLT and Ki-67 immunohistochemistry has been shown [2, 11]. Although full kinetic analysis is, in principle, the most accurate method for determining net uptake of [^18^F]FLT, it is also relatively complex, at the expense of clinical applicability.

Two simplified methods often are used to (semi-)quantitatively assess [^18^F]FLT uptake: graphical (Patlak) analysis [12] and standardised uptake values (SUV). Patlak analysis assumes irreversible trapping in tissue, and its accuracy thus depends on the assumption that no significant dephosphorylation occurs within the time course of the study. Both NLR and Patlak measure net uptake of [^18^F]FLT, taking into account the concentration of tracer in plasma during the course of the study. Only NLR, however, allows for measurements of individual rate constants between compartments and for an implicit correction for blood volume in the tissue of interest. SUV is the ratio of tissue concentration and injected activity at a certain time after administration of the tracer. It does not take tracer kinetics into account but has the advantage that it is a single-scan procedure that does not require plasma data. Previous studies have shown a good correlation between [^18^F]FLT SUV and net uptake calculated using Patlak analysis in for example untreated lung cancer and breast cancer patients, as well as in patients with recurrent glioma [8, 11, 13]. Kenny and co-workers [14] showed that both SUV and Patlak-derived *K*_*i*_ predicted response to chemotherapy for breast cancer after the first cycle of chemotherapy with good reproducibility. They did not specifically address the relationship of changes in SUV with those in NLR-derived *K*_*i*_.

For simplified uptake measures to be valid for monitoring response or predicting outcome, their relationship with the more accurate outcome measures of full kinetic analysis must be similar before and after therapy. Chemotherapy, however, might alter the correlations between NLR, Patlak and SUV, as has previously been shown for 2-deoxy-2-[^18^F]fluoro-D-glucose [15]. This could be due to changes in tumour blood flow, blood volume or plasma clearance of the tracer. The changes are accounted for in full kinetic analysis (NLR), but not in the use of SUV. In those cases, the use of SUV can lead to erroneous conclusions on response or progressive disease. Therefore, the aim of the present study was to validate simplified [^18^F]FLT uptake measures for monitoring response to chemotherapy in locally advanced breast cancer (LABC) by evaluating their relationships with NLR before and after chemotherapy.

## Materials and Methods

### Patients

Data were used from 15 LABC patients participating in an ongoing response monitoring study, the protocol of which was approved by the medical ethics review committee of the VU university medical centre and for which all patients had given written informed consent. Patients underwent an [^18^F]FLT scan shortly before the start of chemotherapy and again 3 weeks later, shortly before the second cycle of chemotherapy in case of traditional, three-weekly schemes (*n* = 12) or before the fourth cycle in case of weekly schemes (*n* = 3). The median delay between baseline PET scan and start of chemotherapy was 1 day (range 0–9 days). Chemotherapy consisted of fluorouracil and cyclophosphamide combined with either epirubicin or doxorubicin.

### PET Acquisition Protocol

PET scans were performed using an ECAT EXACT HR+ scanner (Siemens/CTI, Knoxville, TN). First, a 10-min transmission scan over the tumour area was performed using three retractable ^68^Ge/^68^Ga line sources. A 60-min dynamic emission scan (6 × 5, 6 × 10, 3 × 20, 5 × 30, 5 × 60, 8 × 150, 6 × 300 s) was subsequently performed in 2D acquisition mode after bolus injection of ~370 MBq [^18^F]FLT.

During the [^18^F]FLT scan, six venous samples were drawn at set times, both for immediate measurement of whole blood and plasma radioactivity concentrations and for measurement of metabolite fractions using solid-phase extraction chromatography to separate FLT from FLT-glucuronide. For this procedure 0.3 ml plasma was dissolved in 2 ml water. This solution was brought onto a SepPak® Vac 6-cc (1 g) C18 cartridge (Waters Corporation, Milford, MA). The eluate was collected, after which the cartridge was rinsed with 5 ml water to collect polar metabolites, primarily being [^18^F]FLT-glucuronide. The cartridge was then rinsed with 5 ml ethanol 96% to collect the parent compound. All fractions and the cartridge were counted using a Wallac 1480 Wizard well counter (Perkin-Elmer Life Science, Zaventem, Belgium), and the percentage parent within each plasma sample calculated.

### Image Reconstruction and Processing

Scan data were normalised and corrected for dead time, decay, scattered radiation, random coincidences and photon attenuation, and were reconstructed using filtered back projection (FBP) with a Hanning filter (cut-off 0.5 cycles/pixel). This resulted in a transaxial spatial resolution of approximately 7 mm full width at half maximum (FWHM). For region of interest (ROI) definition, the last three frames (*i.e.*, 45–60 min p.i.) were summed and reconstructed using attenuation-weighted ordered subset expectation maximisation with two iterations and 16 subsets, followed by post smoothing using a 5-mm FWHM Gaussian filter to obtain the same resolution as the dynamic images reconstructed with FBP. Volumes of interest (VOIs) were defined semi-automatically over the tumour by applying a threshold of 70% of the maximum pixel value within the lesion. Tumour VOIs were transferred to the FBP-reconstructed dynamic data to create time–activity curves (TAC). In patients with multiple breast cancer lesions, the primary tumour was analysed. Arterial TACs were measured using 1.5-cm-diameter circular ROIs manually defined over the aortic arch, ascending aorta, left atrium and left ventricle in the frame of the FBP-reconstructed dynamic image in which the injected bolus was best seen passing through these structures [16, 17]. These ROIs were then projected onto all frames. Absolute radioactivity concentration in the arterial TACs was verified using the radioactivity concentrations measured in the blood samples. Arterial TACs were then converted to image-derived input functions by first multiplying them with a single exponential fit to plasma to whole blood ratio data and subsequently with a sigmoid function fit [18] to parent fraction data.

### Data Analysis

Data were analysed using in-house developed software written in Matlab (Natick, MA). The following analytical methods were applied:

1. Net uptake rate (*K*_*i*_) was determined by NLR, using reversible or irreversible two-tissue compartment models with four (NLR_4*k*_) or three (NLR_3*k*_; *k*_4_ = 0) parameters, respectively, and a blood volume parameter. The presence of a fourth rate constant *k*_4_ and the need to include this in the NLR model were assessed by comparing residual sum of squares of fits with and without a *k*_4_ parameter using the Akaike and Schwarz criteria [19].
2. Patlak analysis, giving net uptake rate *K*_*i*_, using the 10–60-min post-injection data,
3. SUV for the 50–60-min interval normalised to body weight, and
4. Tumour-to-whole blood ratio (TBR), *i.e.*, tumour SUV normalised to whole blood SUV at 50–60 min p.i., obtained from the arterial TAC.

The metabolite-corrected plasma time–activity curve was used as input function in both NLR and Patlak analyses. Correlation and agreement between all measures of [^18^F]FLT uptake and NLR were assessed using orthogonal regression, Spearman’s correlation coefficient and intraclass correlation coefficients (ICC). Relative and absolute changes in Patlak-derived *K*_*i*_, SUV and TBR were compared with NLR_3*k*_-derived *K*_*i*_ using orthogonal regression. Confidence intervals of regression parameters were estimated by bootstrapping using 1,000 resamples obtained by random sampling with replacement from the measured data.

## Results

### Patients and Scans

For one patient, [^18^F]FLT scans were excluded because no good fit could be obtained for the second scan, probably due to patient movement. The mean fraction of parent [^18^F]FLT at 60 min p.i. was 79% and 80% for baseline and post-chemotherapy measurements, respectively, with a range of 71–85% at baseline and of 74–84% after chemotherapy. There were no significant differences between pre- and post-therapy values (*p* = 0.68). Mean whole blood and plasma SUV at 60 min p.i. were significantly lower for post-chemotherapy [^18^F]FLT scans than for baseline scans (whole blood: mean (SD) SUV 0.57 (0.09) *versus* 0.61 (0.09), *p* = 0.05, and plasma: mean (SD) SUV 0.66 (0.12) *versus* 0.72 (0.10), *p* = 0.02).

### Data Analysis

The NLR_3*k*_ model provided better fits than the NLR_4*k*_ model in 17 out of 28 (61%) [^18^F]FLT scans, respectively, according to the Akaike criterion, with similar results for the Schwarz criterion. Based on these criteria, a fourth rate constant could not be reliably identified and therefore NLR_3*k*_ was used in the remainder of this study.

**Fig. 1:**
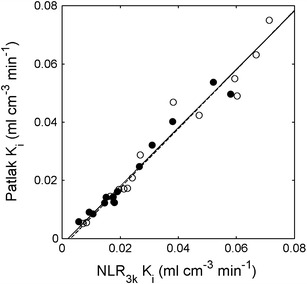
Correlation of Patlak-derived *K*_*i*_ *versus* NLR_3*k*_-derived *K*_*i*_ at baseline (*closed circles, solid line*) and post-chemotherapy (*open circles, dashed line*). The *lines* are orthogonal regressions.

Figure 1 shows net uptake rates of [^18^F]FLT as measured using Patlak analysis *versus* those measured using NLR_3*k*_. Corresponding correlation parameters are shown in Table 1. No significant differences between baseline and post-chemotherapy slopes of the relationships between Patlak and NLR were found.

**Table 1:** [^18^F]FLT simplified measures *versus* NLR

	NLR_3*k*_ *K*_*i*_ Patlak *K*_*i*_	NLR_3*k*_ *K*_*i*_ SUV	NLR_3*k*_ *K*_*i*_ TBR
Spearman’s rho	0.98	0.96	0.96
ICC	0.98	n.a.	n.a.
Slope baseline (CI)	1.01 (0.90–1.13)	101 (90–113)	140 (119–164)
Intercept baseline (CI)	−0.003 (−0.005 to 0.000)	0.34 (−0.00 to 0.75)	0.16 (−0.42 to 0.64)
Slope post-therapy (CI)	1.00 (0.86–1.17)	108 (95–142)	147 (136–179)
Intercept post-therapy (CI)	−0.002 (−0.006 to 0.001)	0.09 (−0.49 to 0.37)	0.15 (−0.41 to 0.41)

*n.a.* not available

**Fig. 2:**
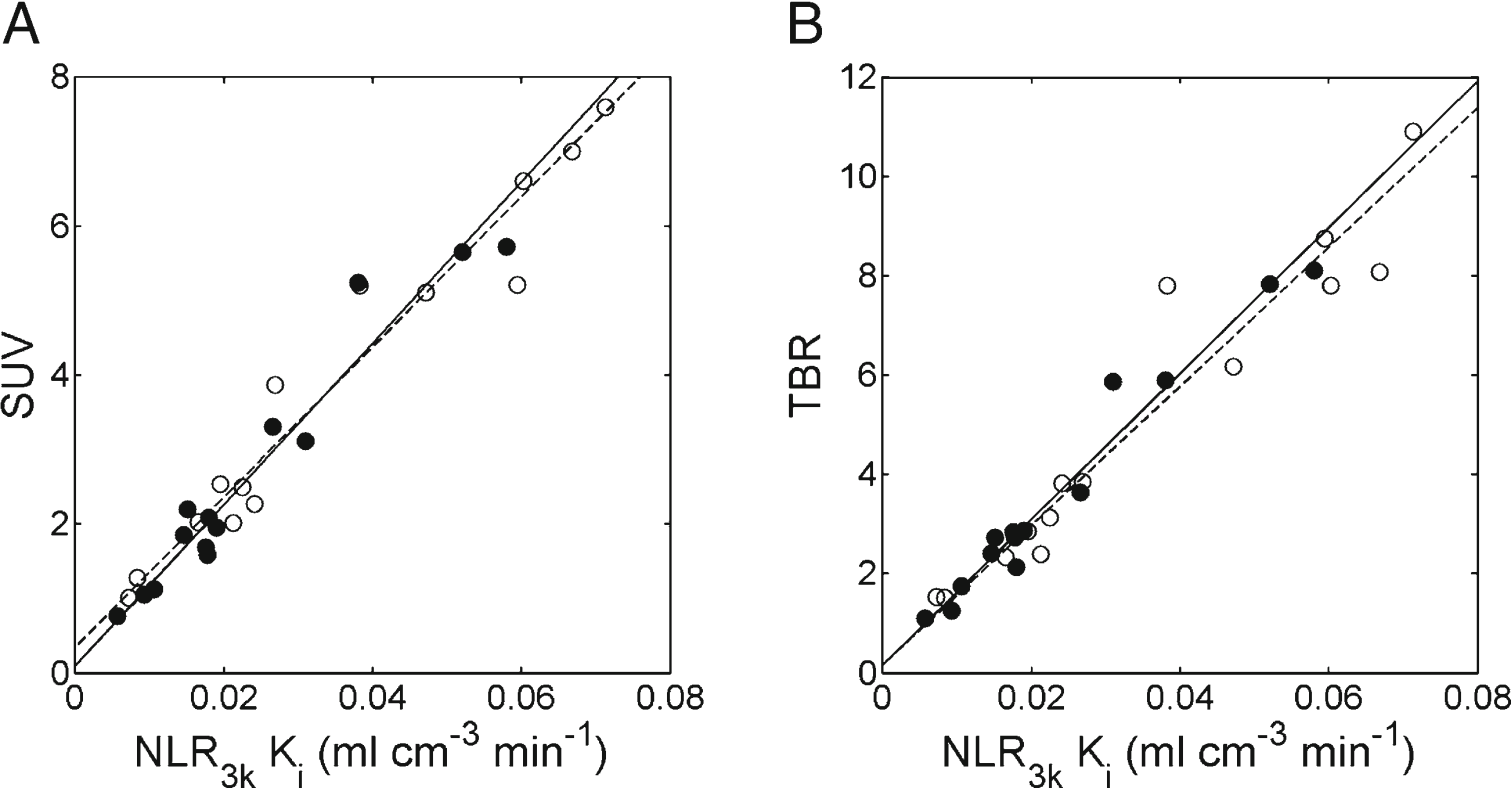
Correlation of **a** SUV and **b** TBR *versus* NLR_3*k*_-derived *K*_*i*_, at baseline (*closed circles, solid line*) and post-chemotherapy (*open circles, dashed line*). The *lines* are orthogonal regressions.

Figure 2 shows correlations between SUV, TBR and NLR_3*k*_-derived *K*_*i*_. Corresponding relationships are summarized in Table 1. Correlation and agreement of SUV and TBR with NLR_3*k*_-derived *K*_*i*_ were similar. A non-significant post-chemotherapy increase in slope between NLR_3*k*_-derived *K*_*i*_ and SUV of about 7% was found.

**Fig. 3:**
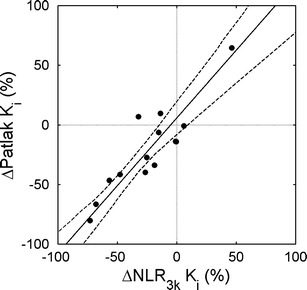
Correlation between relative change in Patlak *K*_*i*_ and NLR_3*k*_ *K*_*i*_. The *solid line* is an orthogonal regression; the *dashed lines* show the 95% confidence interval of the regression line.

**Fig. 4:**
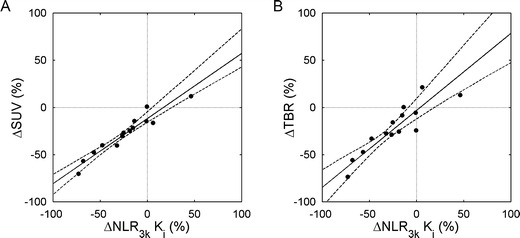
Correlation between relative change in **a** SUV and **b** TBR *versus* NLR_3*k*_ *K*_*i*_. The *solid lines* are orthogonal regressions; the *dashed lines* show the 95% confidence intervals of the regression lines.

Figures 3 and 4 and Table 2 show *relative* changes in simplified uptake measures *versus* those obtained for NLR_3*k*_-derived *K*_*i*_ after chemotherapy. The slope of ΔSUV *versus* Δ*K*_*i*_ was significantly smaller than one (0.69, confidence interval CI 0.57 to 0.88), and a significant negative bias of −0.12 (CI −0.16 to −0.05) in ΔSUV was seen. Absolute changes in SUV showed a significant bias of −0.20 (CI −0.37 to −0.03) as well. The slope of ΔTBR *versus* Δ*K*_*i*_ was not significantly different from one (0.82, CI 0.56–1.13), and bias was smaller and non-significant (−0.03, CI −0.12 to 0.09) as well, although the correlation between ΔTBR and Δ*K*_*i*_ (rho = 0.93) was slightly lower than between ΔSUV and Δ*K*_*i*_ (rho = 0.96).

**Table 2:** [^18^F]FLT: relative change in simplified measures *versus* NLR

	Patlak *K*_*i*_	SUV	TBR
Spearman’s rho	0.81	0.96	0.93
ICC	0.89	0.90	0.88
Slope (CI)	1.14 (0.85–1.39)	0.69 (0.57–0.88)	0.82 (0.56–1.13)
Intercept (CI)	0.06 (−0.08 to 0.20)	−0.12 (−0.16– −0.05)	−0.03 (−0.12 to 0.09)

## Discussion

In the present study, the use of simplified uptake measures for measuring breast cancer treatment response using [^18^F]FLT was compared to non-linear regression, *i.e.*, to full tracer kinetic analysis. Although previous studies have shown a good correlation between [^18^F]FLT SUV and net uptake calculated using NLR and Patlak analysis in untreated lung cancer and breast cancer patients [8, 11], the present study also addresses correlation between responses as measured using NLR and simplified methods. Therapy-induced changes in SUV were negatively biased compared to changes in NLR-derived *K*_*i*_, with no change in *K*_*i*_ corresponding to an 11% decrease in SUV. TBR did not suffer from this bias.

As [^18^F]FLT uptake is primarily mediated by TK-1 activity, it can be argued that *k*_3_ may be a more accurate predictor of tumour proliferation than *K*_*i*_, which is also dependent on other factors (*i.e.*, blood flow). Unfortunately, however, the accuracy of NLR in determining micro parameters such as *k*_3_ is far lower than that of *K*_*i*_ [20], with more than half of the individual *k*_3_ measurements showing standard errors of 20% or more. Since previous studies have shown good correlation between NLR-derived *K*_*i*_ and the proliferation marker Ki-67, as determined by immunohistochemistry [2], this *K*_*i*_ was chosen as the standard in the present study. The use of a fourth parameter has been suggested for [^18^F]FLT based on the potentially reversible behaviour of the tracer [8]. However, apart from the three-parameter model being preferred by the Akaike and Schwartz criteria in the present study, *K*_*i*_ values derived from the reversible model showed a much higher uncertainty than those determined using the irreversible model. In addition, Patlak plots of the 10–60-min interval were linear, which suggests that in the present study no dephosphorylation of [^18^F]FLT occurs during the first 60-min p.i.

As shown previously [5, 10, 21], results of the Patlak analysis correlated very well with NLR_3*k*_. In addition, no differences in pre- and post-chemotherapy relationships with NLR_3*k*_ were found, and there was a high correlation between changes in the Patlak and NLR_3*k*_-derived *K*_*i*_ values. However, as Patlak analysis still requires dynamic scanning and a plasma input function, it is generally not considered to be a measure that can be used in routine clinical practice.

The fact that a significant negative bias in ΔSUV compared to Δ*K*_*i*_ was found for [^18^F]FLT suggests that less tracer was available for uptake into malignant tissue, possibly because of higher post-chemotherapy uptake in other (normal) tissues as a result of the treatment. This is confirmed by a significantly lower plasma radioactivity concentration at 60 min p.i. A reduction in tumour perfusion would cause a decrease in both SUV and *K*_*i*_ and can therefore not explain the preferential reduction of SUV.

Figure 4 suggests that SUV is considerably less sensitive than NLR_3*k*_ in detecting therapy-induced changes in tumour metabolism, with a slope of ΔSUV *versus* Δ*K*_*i*_ of about 0.7. However, previous studies have shown that test–retest variability of [^18^F]FLT SUV is considerably better than that of *K*_*i*_ [14, 20]. In general, an approximately 30% larger change in *K*_*i*_ than in SUV had to be found for it to be considered as a response [20]. The lower sensitivity but better reproducibility of SUV suggests that SUV and *K*_*i*_ have comparable sensitivity in response monitoring, with ΔSUV showing a negative bias.

**Fig. 5:**
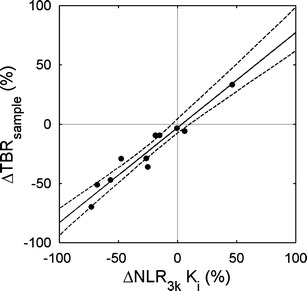
Correlation between relative change in TBR *versus* NLR_3*k*_ *K*_*i*_, using a blood sample at 55–60 min p.i. for calculation of TBR instead of an ascending aorta VOI. The *solid line* is an orthogonal regression; the *dashed lines* show the 95% confidence interval of the regression line.

Potentially, TBR, the change of which does not show a negative bias relative to Δ*K*_*i*_, could be a better measure for treatment response than SUV, provided its test–retest variability is comparable to or better than that of SUV. This needs to be assessed in future studies. The clinical relevance of the differences between SUV and TBR, as found in the present work, will be assessed in a clinical study. In the present work, TBR was calculated using the mean value of the arterial TAC between 50 and 60 min. In 11 out of 14 patients, a venous whole blood sample taken between 55 and 60 min p.i. was available. For these 11 patients, correlation and agreement between ΔTBR and Δ*K*_*i*_ were better when TBR was based on blood sample data than on image-derived blood data (Pearson’s *r* ^2^ 0.92 *versus* 0.81; Spearman’s rho 0.95 for both cases), whilst the relation between ΔSUV or image-based ΔTBR and Δ*K*_*i*_ was similar for these 11 patients as for the complete group of 14 patients (Fig. 5). This suggests that, probably because of the noisy nature of the arterial TAC at 50–60 min p.i., use of a blood sample might produce more robust TBR values. Although TBR, as opposed to SUV, does take post-therapy changes in whole blood clearance into account, it cannot account for therapy-induced changes in metabolism. If major changes in metabolism are observed for a certain type of chemotherapy, the validity of TBR has to be addressed by comparison to full kinetic modelling using NLR with a metabolite-corrected plasma input function, and full kinetic modelling may be preferable.

## Conclusion

For [^18^F]FLT, change in SUV was negatively biased compared to change in NLR_3*k*_-derived *K*_*i*_, with no change in *K*_*i*_ corresponding to a significant decrease in SUV. Use of TBR did not show this bias and has a similar correlation to NLR_3*k*_-derived *K*_*i*_. Therefore, tumour-to-whole blood ratio may be preferred to SUV as a simplified measure for monitoring response to chemotherapy in LABC when using [^18^F]FLT.
